# Os iliaque en nid d'abeille

**DOI:** 10.11604/pamj.2013.16.95.3514

**Published:** 2013-11-12

**Authors:** Rhizlane Berrady, Wafaa Bono

**Affiliations:** 1 CHU Hassan II, Faculté de médecine de Fès, Service de Médecine Interne, Fès, Maroc

**Keywords:** Os iliaque, nid d'abeille, hydatidose, hipbone, Honeycomb, hydatidosis

## Image en medicine

L'hydatidose est une anthropozoonose due au développement de la forme larvaire du ténia Echinococcus granulosus chez l'homme. L'infestation humaine est accidentelle. Elle atteint le foie et le poumon dans 85 à 90% des cas et l'os dans seulement 0.5 à 2% des cas. Du fait de sa longue évolution, l'hydatidose osseuse est généralement découverte chez l'adulte. La douleur et la tuméfaction sont les principaux signes d'appel. Les tests immunologiques qualitatifs et quantitatifs sont d'un grand apport diagnostique mais ne sont positifs que dans 30 à 40% des cas. Quel que soit son siège, l'hydatidose se traduit sur les radiographies par une ostéolyse lacunaire en “nid d'abeille” avec un cortical longtemps respecté. L'ostéocondensation est absente ou discrète sauf en cas de surinfection, absence de réaction périostée ou de reconstruction osseuse. La forme générale de l'os est conservée. Nous rapportons le cas d'un patient âgé de 20 ans, suivi pour douleur chronique de la hanche droite avec installation de boiterie. La radiographie standard faite objective une destruction de l'articulation coxo-fémorale avec des lésions multiples en nid d'abeille de l'os iliaque, et respect de la forme générale de l'os. Une biopsie osseuse faite revient en faveur d'un kyste hydatique intra-osseux sans signe de malignité. La sérologie hydatique est revenue positif avec un titre de 1/2560. Le patient est mis sous traitement médical à base d'Albendazole.

**Figure 1:**
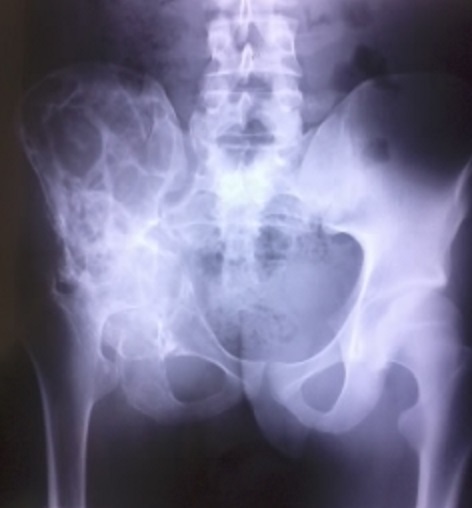
Destruction de l'articulation coxo-fémorale avec des lésions multiples en nid d'abeille de l'os iliaque

